# Genotyping the *GALNT14* gene by joint analysis of two linked single nucleotide polymorphisms using liver tissues for clinical and geographical comparisons

**DOI:** 10.3892/ol.2014.2507

**Published:** 2014-09-05

**Authors:** KUNG-HAO LIANG, PEI-CHING YANG, CHAU-TING YEH

**Affiliations:** 1 Liver Research Center, Chang Gung Memorial Hospital, Taipei 10507, Taiwan, R.O.C.; 2 Molecular Medicine Research Center, Chang Gung University, Taoyuan 10507, Taiwan, R.O.C.

**Keywords:** glycosyltransferase, hepatocellular carcinoma, single nucleotide polymorphism, non-viral etiology, restriction enzyme

## Abstract

A *GALNT14* single nucleotide polymorphism, rs9679162, has recently been found to be capable of predicting chemotherapy responses in patients with far-advanced hepatocellular carcinoma (HCC). In the present study, a novel assay was designed and genotyping was performed on 244 surgically removed liver tissues. This assay employed two polymerase chain reaction (PCR)-generated restriction enzyme sites to simultaneously determine the genotypes of two adjacent single nucleotide polymorphisms (SNPs), rs9679162 and rs6752303, on the *GALNT14* gene. Genotypes determined by this assay reached 100% concordance with those detected by the direct sequencing method. Clinical analysis showed that the TT genotype of rs9679162 was lower in percentage among patients with virus-originated HCC compared with those with non-viral HCC (22.57 vs. 47.06%, respectively; P=0.023). The proportion of the TT genotype in the 244 HCC patients (24.18%) did not deviate significantly from those of two public-domain (HapMap) Chinese cohorts from Denver, Colorado, USA (28.44%) and Beijing, China (30.15%) (P>0.05). The proportion of the TT genotype was significantly higher in Japanese and African populations (42.11–54.55%; P<0.0001) but significantly lower in an Italian cohort (7.84%; P=0.0004). In conclusion, the novel PCR-generated double restriction enzyme sites method could correctly determine the genotypes of two target SNPs in *GALNT14* in liver tissues. The TT genotype was associated with the non-viral etiology of HCC. A marked variation in ethnicity was found for the distribution of this genotype.

## Introduction

As an aggressive cancer, hepatocellular carcinoma (HCC) is the third leading cause of cancer-related mortality and the fifth most common solid malignant tumor (1). Early-stage HCC can be treated by surgical resection, while late-stage HCC patients are often treated by sorafenib or combination chemotherapy (2). Viral infection is the major cause of HCC; in Asia, hepatitis B virus (HBV) is the predominant cause, while in the Western world, hepatitis C virus (HCV) is the major cause (3).

Recently, a single nucleotide polymorphism (SNP) on *GALNT14* has been repetitively shown to correlate with the therapeutic responses of combination chemotherapy in independent cohorts of patients with far-advanced HCC, and the TT genotype of the SNP, rs9679162, was correlated with a good post-chemotherapy prognosis (2,4). The genotyping technique in these studies followed the traditional concept, using peripheral blood cells to provide chromosomal DNA and polymerase chain reaction followed by direct sequencing to determine genotypes. To facilitate future studies regarding the association between this genotype and other clinical parameters, a more convenient assay was necessary. The assay required the capability to process one or a few samples (in cases where a few patients were to be assessed per day, for clinical purposes) at low costs, while providing accurate results promptly. The assay required the capability to use tissue-derived chromosomal DNA for genotyping for retrospective studies. Additionally, it should be able to be conducted in medical facilities lacking sequencing machines, as HCC is prevalent in a number of third world countries (5).

The identification of restriction enzymes capable of recognizing and cleaving DNA at specific sites has been a cornerstone of modern biotechnology (6). Genomic DNA digested by restriction enzymes becomes DNA fragments of varied lengths, creating a personalized signature called restriction fragment length polymorphisms (RFLPs). Prior to the widespread use of high-throughput sequencing and genotyping methods, RFLP was one of the major assays for pinpointing genomic regions responsible for various phenotypic traits (7). This technology has led toward the discovery of the *CFTR* gene, the first disease-bearing gene ever identified by positional cloning (8,9). The method has also been used in various clinical assays, including the diagnosis of sickle cell anemia (10).

A variety of SNP assays, including the TaqMan and fluorescence polarization assays, have also been developed (11). One shared characteristic of these assays is the requirement of batches of samples for providing large enough numbers of signals for each of the three genotypes. The signals are then used to delineate the genotype-specific intensity distribution ‘on-the-fly’, in other words, an unsupervised base-calling technique. Such a platform was found to be suitable in the validation stage for handling a large number of pre-collected samples, however, it was not found to be practical for clinical use considering the daily fluctuations of patient numbers (12). To prepare for future clinical use, a practical, low-cost assay that could be used in a small hospital of a remote village, as well as in large urban medical centers, was developed in the present study. The assay was performed on surgically resected liver tissues, and the derived *GALNT14* genotypes were correlated with the clinical data of the HCC patients. Finally, the geographical distributions of the genotypes were examined.

## Materials and methods

### Patients and clinical data

This study was conducted under the approval of the Institutional Review Board of Chang Gung Memorial Hospital, Taiwan. All study subjects were adults and provided written informed consent. A total of 244 patients with HCC treated by surgical resection were included, and their surgical specimens were retrieved from the Tissue Bank of Chang Gung Medical Center. Samples were obtained from the non-tumorous sections of the surgical specimens. All samples were frozen at −70°C immediately after surgical resection, until use. HBV was diagnosed if the HBV surface antigen was detected in the patient’s peripheral blood. HCV was diagnosed if anti-HCV antibody was detected.

### Design of the polymerase chain reaction (PCR)-generated double restriction enzyme sites-RFLP assay

The basic concept behind the proposed assay was to incorporate the target bi-allelic SNP as part of an artificially-introduced restriction enzyme cutting site. Together with adjacent nucleotide bases, one allele of the SNP could constitute a restriction enzyme recognizable sequence, while the other allele could not. As a consequence, samples with distinct SNP alleles manifested as distinct length polymorphisms following restriction enzyme digestion.

The genetic engineering method was employed to introduce sequence signatures artificially. The assay was based on nested PCR. A first-step PCR was designed to amplify the DNA fragment containing the target SNPs without any naturally occurring restriction enzyme cutting sites. The second-step PCR employed a set of specially designed primers targeted to the first amplicons to introduce desired sequence signatures, which were recognizable by restriction enzymes for allele-specific cuttings.

**Figure 1:**
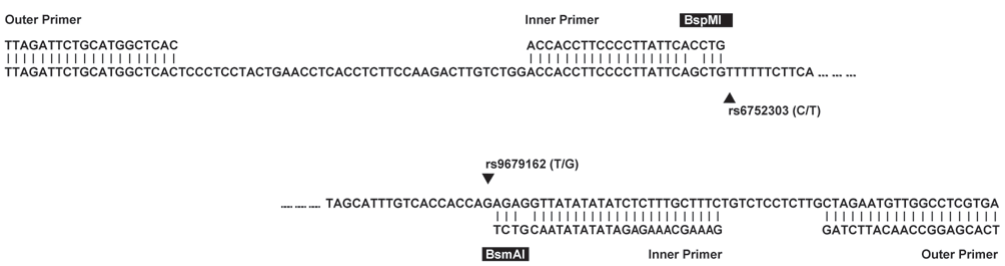
Polymerase chain reaction (PCR)-generated double restriction enzyme sites genotyping assay. The central sequence was the reference genomic DNA sequence. This assay was a nested PCR assay where two primer sets, the outer and inner primers, were used. Each of the inner primers had one base mismatch to the reference sequence for creating a restriction site. The cutting site of *Bsm*AI (GTCTC) was created if the allele type on rs9679162 was G (corresponding to C in the other strand). The cutting site of *Bsp*MI (ACCTGC) was created if the allele type on rs6752303 was C.

Accordingly, an assay was designed to simultaneously genotype two adjacent and tightly linked SNPs in the *GALNT14* gene; each was shown to correlate with the chemotherapy responses of patients with far-advanced HCC (4). *GALNT14* resides on chromosome 2, and the two SNPs, rs9679162 and rs6752303, are in the intronic region of the gene. A set of outer primers was designed to amplify a 172-base amplicon containing the two SNPs (Table I). No endogenous restriction sites were found in the amplicon. The inner primers were then used to introduce two cutting sites of *Bsm*AI and *Bsp*MI (GTCTC and ACCTGC respectively), which were formed partly by the primer and partly by rs9679162 and rs6752303 (Fig. 1). The second amplicon had a length of 80 bases. The allele types at the site of the SNP were essential in determining whether the cutting could proceed, resulting in fragments (~55 bases). The cut and uncut fragments manifested as lower and upper bands in the gel image of electrophoresis respectively. A look-up table (Table II) described how genotypes can be defined by the band patterns.

### Genomic DNA preparation and the first PCR amplification

Genomic DNA was extracted from clinical samples using QIAamp^®^ DNA Mini and Blood Mini kits (Qiagen, Düsseldorf, Germany). DNA amplification was performed in PCR reaction mixture consisting of genomic DNA (5 μl), Taq DNA polymerase 2.0 Master Mix Red (MgCl_2_, 1.5 mM; 25 μl; Ampliqon, Glostrup, Denmark), the first set of primers (10 μM, 0.25 μl each; Table I) and water (19.5 μl). PCR was carried out for 35 cycles under the following conditions: Initial denaturation at 94°C for 5 min, denaturation at 94°C for 1 min, annealing at 55°C for 1 min and extension at 72°C for 1 min, followed by a final extension step at 72°C for 10 min.

### Second PCR amplification

The reaction mixture of the second PCR amplification comprised the first PCR amplicon (i.e., the product of the previous step; 0.1 μl), Taq DNA polymerase 2.0 Master Mix Red (MgCl_2_, 1.5 mM; 25 μl), the second set of primers (10 μM, 0.25 μl each; Table I), and water (24.5 μl). PCR was performed for 20 cycles under the following conditions: Initial denaturation at 94°C for 5 min, denaturation at 94°C for 1 min, annealing at 55°C for 1 min and extension at 72°C for 1 min, followed by a final extension step at 72°C for 10 min.

### Restriction enzyme digestion and gel electrophoresis

The samples were then digested by restriction enzymes. The reaction mixture comprised the second amplicon (3 μl), 10X buffer (2 μl), restriction enzymes *Bsm*AI or *Bsp*MI (1 μl) (New England Biolab, Ipswich, MA, USA) and water (14 μl). Incubation temperatures were 55°C for *Bsm*AI and 37°C for *Bsp*MI. The incubation time was 2 h. The lengths of the DNA fragments were analyzed by a subsequent electrophoresis. This was performed by loading the *Bsm*AI- or *Bsp*MI-treated samples (15 μl) into wells of 2.5% superfine resolution™ agarose gel (Amresco, Solon, OH, USA), using 50 V for 60 min. Genotypes were then defined based on the gel image.

### Evaluation of assay accuracy

Sanger sequencing was performed to the first amplicon. The sequencing-derived genotypes were compared with those obtained by the proposed assay.

### Statistical and data analysis

The genotypes were correlated with the clinical data of the subjects. χ^2^ tests were used to compare the categorical variables, including the genotype, gender and presence of cirrhosis, with respect to the TT and non-TT genotypes. Comparisons of numerical variables were performed by two sample t-tests assuming unequal variance. All P-values reported here are two-tailed. SPSS 12.0 software (SPSS, Inc., Chicago, IL, USA) was used for the aforementioned analysis. The public domain HapMap genotype counts were obtained from the official website (http://hapmap.ncbi.nlm.nih.gov/) on Oct 11, 2013, in order to facilitate the analysis (13). The Wellcome Trust Case-Control Consortium (WTCCC) data were downloaded from the European Genome-phenome Archive (EGA) at https://www.ebi.ac.uk/ega/ with permission from the WTCCC (14).

## Results

**Figure 2:**
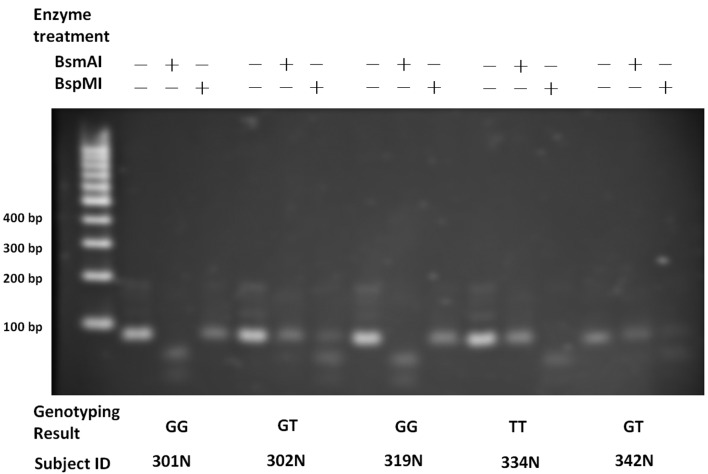
Example gel images of the proposed assay. Genotypes were indicated by the appearance of upper and lower bands according to Table II.

The genotypes of rs9679162 and rs6752303 obtained from the proposed assay and the conventional Sanger sequencing were identical, reaching a concordance rate of 100% in the 244 subjects. Fig. 2 shows examples of the gel images of five subjects. Homozygous and heterozygous genotypes were manifested as different band patterns. The genotypes of the two adjacent SNPs were highly associated (linkage disequilibrium, r^2^=0.984).

The HCC subjects were of either viral (HBV and/or HCV) or non-viral etiologies (Table III). By comparing the clinical data, it was found that the TT type was under-represented in the viral subgroup in comparison with the non-viral subgroup (P=0.0231). A subsequent stratification of the viral groups showed that the HBV and HCV subgroups had a higher percentage of the non-TT genotype compared with the non-viral HCC subgroup (P=0.0268 and P=0.0331, respectively). No other associations between genotypes and clinical parameters were observed (Table III). The viral/non-viral etiology did not correlate with a history of alcoholism (P=0.750). No significant deviations from the Hardy-Weinberg equilibrium were found in either the viral or non-viral HCC subgroups (Table IV).

Geographical distributions of the genotypes were conducted by comparison between the present data and the HapMap data in the public domain (Table IV). The proportion of the TT genotype in the patients with HCC in the present study (24.18%) did not deviate significantly from those of two Chinese cohorts from Denver, Colorado, USA (28.44%) and Beijing, China (30.15%) (P>0.05 for each). The proportion of the TT genotype was significantly higher in Japanese populations from Tokyo (46.02%) and in three African populations (Kenya, 54.55%; Nigeria, 48.30%; and African ancestry in USA, 42.11%) (all P<0.0001). Notably, the proportions were significantly lower in cohorts of European descendants, including the Toscani, Italian cohort (7.84%; P=0.0004), Utah residents with Northern and Western European ancestry (12.39%; P=0.0097), the British 1958 birth cohort (15.76%; P=0.0010) and the UK National blood service cohort (17.01%; P=0.0063).

## Discussion

Reliable genetic biomarkers for patients with HCC could offer critical information for personal preventive and therapeutic strategies, and GALNT14-rs9679162 has been shown to have such a potential (2,4). The GALNT family of glycosyltransferases has long been indicated to be involved in the onset and progression of various cancers, including HCC, although the molecular mechanisms remain largely elusive (15–19). *GALNT14* has been shown to be involved in the glycosylation of multiple cellular substrates, including death receptors, DR4 and DR5 (20). The mRNA level has been shown to positively correlate with cancer cell sensitivity to DR4 and DR5 agonists (20,21). It has also been found that GALNT14 proteins are more abundant in breast carcinoma compared with normal tissues, but that the expression levels decrease in more advanced stages of cancer (22).

In the present study, a comparison of genotype distributions of various HCC etiologies revealed that the TT genotype, previously reported to indicate a good post-chemotherapy prognosis, was present in a smaller proportion in the viral HCC subgroup (22.57%) compared with the non-viral HCC subgroup (47.06%, P=0.0231). The reduced percentage of the TT genotype in the viral HCC subgroup suggested that it may impart a lower risk of HCC among chronic HBV and HCV patients. As the TT genotype was associated with a good chemotherapy response, we hypothesized that HCC cells possessing this genotype were more susceptible to chemotherapy agents owing to the link between the GALNT14 and apoptosis pathways (20). As such, the hepatocytes of TT genotype could be more vulnerable under HBV or HCV infection and thus, less easily progress to liver cancer. Such a protective role appeared to apply to HBV and HCV, as the two subgroups had reduced TT percentages.

Comparing the current study to the public-domain HapMap data, it was found that the Japanese population had a higher percentage of the TT genotype (Table IV). Notably, Japanese patients with advanced HCC have been reported to have high response rates to interferon and 5-fluorouracil combination therapy (23). We hypothesize that the high proportion of the TT genotype in Japanese patients may underlie the high response rates in this country.

In summary, the current study presented a novel genotyping method through the use of PCR-generated double restriction enzyme sites. This method could correctly genotype two linked SNPs in *GALNT14*. Additionally, the TT percentage of rs9679162 was lower in the viral subgroups of HCC.

## Figures and Tables

**Table I:** Primer sequences for the restriction fragment length polymorphism genotyping assays.

Primer sequence	Tm,°C
Outer primer	
TCACGAGGCCAACATTCTAG	58.9
TTAGATTCTGCATGGCTCAC	56.4
Inner primer	
GAAAGCAAAGAGATATATATAACGTCT	59.2
ACCACCTTCCCCTTATTCACCTG	66.2

Tm, temperature.

**Table II:** A look-up table for interpreting the band patterns of the proposed assay.

A, rs9679162				

		Genotype^a^		
		
RE	Band	TT (AA)	TG (AC)	GG (CC)
*Bsm*AI cut	Upper	+	+	−
	Lower	−	+	+

B, rs6752303				

		Genotype^a^		
		
RE	Band	CC (GG)	CT (GA)	TT (AA)

*Bsp*MI cut	Upper	−	+	+
	Lower	+	+	−

a Genotypes in parentheses are bases in the antisense strand.

RE, restriction enzyme.

**Table III:** Clinical parameters of early-stage HCC patients.

Parameter	rs9679162 TT	rs9679162 non-TT	P-value
Patient number	59	185	
Age, years	54.54±15.04	53.94±14.37	0.786
Male gender	48 (81.36)	147 (79.46)	0.853
Etiology			
HBV	47 (79.66)	158 (85.41)	0.253
HCV	9 (15.25)	36 (19.46)	0.458
HBV-HCV co-infection	5 (8.47)	19 (10.27)	0.678
Non-viral	8 (13.56)	9 (4.86)	0.023
Alcoholism history	25 (42.37)	57 (30.81)	0.331
Cirrhosis	35 (59.32)	99 (53.51)	0.548
Ascities	5 (8.47)	16 (8.65)	0.955
Biochemical analysis			
AFP, n/mL	1876.54±6735.52	10104.53±49077.56	0.054
Albumin, g/dL	3.93±0.65	3.92±0.65	0.935
Bilirubin, mg/dL	1.68±2.94	1.20±1.16	0.224
Creatinine, mg/dL	1.18±0.66	1.25±1.41	0.583
AST, U/l	82.09±77.79	80.63±115.76	0.914
ALT, U/l	76.60±79.00	79.95±108.51	0.804
Prothrombin time, sec	12.24±1.34	12.95±8.08	0.259

Categorical data is presented as n (%) and compared by Pearson’s χ^2^ test. Quantitative data is presented as the mean ± standard deviation and compared by two sample t-test assuming unequal variance. HBV, hepatitis B virus; HCV, hepatitis C virus; AFP, α-fetoprotein; AST, aspartate aminotransferase; ALT, alanine aminotransferase.

**Table IV:** Proportions of rs9679162 genotype in various subgroups.

Subgroups	TT, %	Non-TT, %	Subgroup subject number	Current study vs. public data	HWE P-value
HCC					
Viral	22.57	77.43	226		0.5659
Non-viral	47.06	52.94	17		0.6446
Total	24.18	75.82	244		0.7790
HapMap					
Luhya in Webuye, Kenya	54.55	45.45	110	<0.0001	0.0471
Yoruba in Ibadan, Nigeria	48.30	51.70	147	<0.0001	0.8995
Japanese in Tokyo, Japan	46.02	53.98	113	<0.0001	0.9959
African ancestry in Southwest USA	42.11	57.89	57	<0.0001	0.9999
Mexican ancestry in Los Angeles, California	36.21	63.79	58	0.0647	0.6739
Han Chinese in Beijing, China	30.15	69.85	136	0.2138	0.7203
Chinese in Metropolitan Denver, Colorado	28.44	71.56	109	0.4081	0.9005
Maasai in Kinyawa, Kenya	25.00	75.00	156	0.8705	0.9873
Gujarati Indians in Houston, Texas	16.83	83.17	101	0.1294	0.7804
Utah residents with Northern and Western European ancestry from the Centre d’Etude du Polymorphisme Humain collection	12.39	87.61	113	0.0097	0.4639
Toscani in Italia	7.84	92.16	102	0.0004	0.0927
WTCCC					
British 1958 birth cohort	15.76	84.24	1504	0.0010	0.3033
UK national blood service	17.01	82.99	1499	0.0063	0.9470

a HWE, Hardy-Weinberg equilibrium; WTCCC, The Wellcome Trust Case-Control Consortium.

## References

[1] Ferlay J, Shin HR, Bray F (2010). Estimates of worldwide burden of cancer in 2008: GLOBOCAN 2008. Int J Cancer.

[2] Yeh CT, Liang KH, Lin CC, Chang ML, Hsu CL, Hung CF (2014). A single nucleotide polymorphism on the GALNT14 gene as an effective predictor of response to chemotherapy in advanced hepatocellular carcinoma. Int J Cancer.

[3] Perz JF, Armstrong GL, Farrington LA (2006). The contributions of hepatitis B virus and hepatitis C virus infections to cirrhosis and primary liver cancer worldwide. J Hepatol.

[4] Liang KH, Lin CC, Yeh CT (2011). GALNT14 SNP as a potential predictor of response to combination chemotherapy using 5-FU, mitoxantrone and cisplatin in advanced HCC. Pharmacogenomics.

[5] Venook AP, Papandreou C, Furuse J, de Guevara LL (2010). The incidence and epidemiology of hepatocellular carcinoma: a global and regional perspective. Oncologist.

[6] Roberts RJ (2005). How restriction enzymes became the workhorses of molecular biology. Proc Natl Acad Sci USA.

[7] Botstein D, White RL, Skolnick M, Davis RW (1980). Construction of a genetic linkage map in man using restriction fragment length polymorphisms. Am J Hum Genet.

[8] Rommens JM, Iannuzzi MC, Kerem B (1989). Identification of the cystic fibrosis gene: chromosome walking and jumping. Science.

[9] Collins FS (1992). Positional cloning: let’s not call it reverse anymore. Nat Genet.

[10] Saiki RK, Scharf S, Faloona F (1985). Enzymatic amplification of beta-globin genomic sequences and restriction site analysis for diagnosis of sickle cell anemia. Science.

[11] Kwok PY (2002). SNP genotyping with fluorescence polarization detection. Hum Mutat.

[12] Liang KH, Fen JJ, Chang HH, Wang HW, Hwang Y (2011). A base-calling algorithm for Tm-shifted melting curve SNP assay. J Clin Bioinforma.

[13] Altshuler DM, Gibbs RA, Peltonen L, International HapMap 3 Consortium (2010). Integrating common and rare genetic variation in diverse human populations. Nature.

[14] Wellcome Trust Case Control Consortium (2007). Genome-wide association study of 14,000 cases of seven common diseases and 3,000 shared controls. Nature.

[15] Bauer CH, Vischer P, Grünholz H, Reutter W (1977). Glycosyltransferases and glycosidases in Morris hepatomas. Cancer Res.

[16] Mandel U, Hassan H, Therkildsen MH, Rygaard J, Jakobsen MH, Juhl BR, Dabelsteen E, Clausen H (1999). Expression of polypeptide GalNAc-transferases in stratified epithelia and squamous cell carcinomas: immunohistological evaluation using monoclonal antibodies to three members of the GalNAc-transferase family. Glycobiology.

[17] Kohsaki T, Nishimori I, Nakayama H, Miyazaki E, Enzan H, Nomoto M, Hollingsworth MA, Onishi S (2000). Expression of UDP-GalNAc: polypeptide N-acetylgalactosaminyltransferase isozymes T1 and T2 in human colorectal cancer. J Gastroenterol.

[18] Wu YM, Liu CH, Hu RH, Huang MJ, Lee JJ, Chen CH, Huang J, Lai HS, Lee PH, Hsu WM, Huang HC, Huang MC (2011). Mucin glycosylating enzyme GALNT2 regulates the malignant character of hepatocellular carcinoma by modifying the EGF receptor. Cancer Res.

[19] Park JH, Nishidate T, Kijima K, Ohashi T, Takegawa K, Fujikane T, Hirata K, Nakamura Y, Katagiri T (2010). Critical roles of mucin 1 glycosylation by transactivated polypeptide N-acetylgalactosaminyltransferase 6 in mammary carcinogenesis. Cancer Res.

[20] Wagner KW, Punnoose EA, Januario T (2007). Death-receptor O-glycosylation controls tumor-cell sensitivity to the proapoptotic ligand Apo2L/TRAIL. Nat Med.

[21] Thorburn A, Behbakht K, Ford H (2008). TRAIL receptor-targeted therapeutics: resistance mechanisms and strategies to avoid them. Drug Resist Updat.

[22] Wu C, Guo X, Wang W (2010). N-Acetylgalactosaminyltransferase-14 as a potential biomarker for breast cancer by immunohistochemistry. BMC Cancer.

[23] Obi S, Yoshida H, Toune R, Unuma T, Kanda M, Sato S, Tateishi R, Teratani T, Shiina S, Omata M (2006). Combination therapy of intraarterial 5-fluorouracil and systemic interferon-alpha for advanced hepatocellular carcinoma with portal venous invasion. Cancer.

